# Demographics and Psychological Factors Associated with Adiposity in Nurses

**DOI:** 10.3390/ijerph15040634

**Published:** 2018-03-30

**Authors:** Bernarda Sánchez-Jiménez, Reyna Sámano, Daniela Chinchilla-Ochoa, Rosa Morales-Hernández, Ana Rodríguez-Ventura

**Affiliations:** 1 Sub-Direction of Research in Communitarian Interventions National, Institute of Perinatology, Urales # 800, Col. Lomas de Virreyes, Delegación Miguel Hidalgo, Mexico City C.P. 11000, Mexico; emiberna20@yahoo.com.mx; 2 Department of Nutrition and Bioprogramming, Research Direction, National Institute of Perinatology, Montes Urales # 800, Col. Lomas de Virreyes, Delegación Miguel Hidalgo, Mexico City C.P. 11000, Mexico; ssmr0119@yahoo.com.mx (R.S.); rmh080868@yahoo.com (R.M.-H.); 3 Departament of Neurosciences, National Institute of Perinatology, Urales # 800, Col. Lomas de Virreyes, Delegación Miguel Hidalgo, Mexico City C.P.11000, Mexico; danielachinchilla87@gmail.com

**Keywords:** adiposity, overweight, obesity, nurses, lifestyle, self-esteem, emotional distress

## Abstract

Adiposity-based chronic disease (ABCD), overweight-Ow- or obesity-Ob-) in health personnel is as frequent as in the general population, even though they understand well the importance of maintaining a healthy weight. Thus, it is highly likely that certain demographic and psychological conditions, independently of knowledge, are contributing to develop ABCD. The aim of this study was to examine the association between these factors and ABCD in nurses. Data were collected from a cross-sectional study conducted in a tertiary level institute in Mexico City from 2012 to 2013. All the nurses of the institute of any age, shift, service area and seniority were invited to participate and 55% (265) accepted. We found that ABCD was present in 79.6%, and low self-esteem and emotional distress in 26% and 10%, respectively. Working in the night shift (*p* = 0.031), labor seniority ≥15 years (*p* = 0.006), having 1 or more children (*p* = 0.021) and sessions of physical activity <30 min (*p* = 0.03) were associated with ABCD. Low self-esteem (OR = 2, 95% CI 1.150–3.07, *p* = 0.023) and emotional distress (OR = 4, 95% CI 1.472–13.078, *p* = 0.012) were associated with unhealthy lifestyle (less of 3 days per week and/or less of 30 min per session of physical activity and poor dietary habits). Therefore, strategies to prevent and treat ABCD must consider each context among nurses and psychological disorders need be identified to avoid an unhealthy lifestyle.

## 1. Introduction

Women are the main health carers in families and most of the nursing staff is still female as a result of the process of sex stereotyping of the roles that tend to be transmitted through the socialization process [1]. Nurses educate, care, advise and investigate, but there are no particular programs to care for their health. Some authors have commented that nurses do not display ideal self-concern about their health because they live a double sense of duty and are affected by their environment and job [2]. Additionally, although they have enough self-care knowledge, they often do not have a correct self-care [3]. Health care employees, including nurses, are at increased risk of non-communicable diseases (NCDs) like diabetes, hypertension and coronary heart diseases [4]. The main risks of NCDs are physical inactivity, poor dietary habits, smoking and alcohol abuse. NCD risk factors such as physical inactivity and adiposity based chronic disease (ABCD) have been widely reported among nurses in countries like Australia, United Kingdom, New Zealand and South Africa [2,4,5,6]. On the other hand, nurses experience high levels of stress/distress due to the nature of their work and workplaces; and their socialization into ways of working that minimizes the likelihood of self-care, so then it is also important to evaluate their self-esteem and emotional distress [7,8]. Given all this evidence, we hypothesized that some demographic conditions, such as age, labor seniority, civil state, shift, etc., as well as psychological disorders may be associated with ABCD among nurses. The aim of this study was to identify the frequency of adiposity in nurses and its association with demographic and psychological conditions, besides lifestyle.

## 2. Materials and Methods

### 2.1. Study Design

Data were collected from a cross-sectional study conducted in 2012–2015. During this period, 478 nurses were invited, but only 265 (55%) accepted to participate. Inclusion criteria were: female gender, any age, any labor seniority, any shift and area of service. An exclusion criterion was male gender. Men nurses also participated to avoid discrimination, but their results were not analyzed for this study. This project was approved by the Research and Ethics Committee of our institution, 212250-08361. Written informed consent was obtained from all participants.

### 2.2. Body Mass Index (BMI) and Classification of Overweight (Ow) and Obesity (Ob)

BMI was calculated as the weight divided by height squared (kg/m^2^) and Ow/Ob were classified according to World Health Organization cut-off points [9]. Ow was defined as BMI ≥25 and Ob ≥30.

### 2.3. Demographic and Lifestyle Factors

Age, labor seniority, area of service, shift, civil status and number of children were determined by a questionnaire. We also recorded time per session of physical activity, sessions per week and quality of food through an internal validated questionnaire.

### 2.4. Metabolic Disturbs

Hyperglycemia was considered if fasting glucose was ≥100 mg/dL [10]. Metabolic syndrome was defined according the National Cholesterol Education Program-Third Adult Treatment Panel (NCEP-ATPIII) [11] with three or more of the following characteristics: fasting plasma glucose ≥100 mg/dL, blood pressure ≥130/≥85 mmHg, triglycerides ≥150 mg/dL, HDL <50 mg/dL and/or waist ≥88 cm.

### 2.5. Psychological Evaluation

Self-esteem is a state which the person assumes herself/himself as a competent person. According to Cooper Smith, self-esteem is self-evaluation and/or self-judgment about his/her values. There is an interaction between the person’s self-esteem and ability to understand his/her abilities, in such a way that decreasing self-esteem causes weakness and inability, while increasing self-esteem can revive the feeling of power and value in the person [12]. Emotional distress is defined as the emotional variations that regulate the behavior related to external event, it was measured using Goldberg test, already validated [13,14].

### 2.6. Statistical Analysis

Descriptive statistics was used. *X*^2^ and Student *t*-test were used for categorical and numerical variables, respectively. A multivariable logistic regression model was performed to derive odds ratios (ORs), 95% confidence intervals (95% CIs), and *p*-values. A *p*-value less than 0.05 was considered statistically significant. All statistical analyses were performed using IBM SPSS Statistics version 22 (IBM Inc., Armonk, NY, USA).

## 3. Results

Mean age was 41.5 ± 8.1 years and labor seniority was 18.6 ± 8.5 years, 83% had studies until bachelor or technician level, 64% worked in intensive care areas. Adiposity was present in 79.6%: Ow in 43.4% and Ob in 36.2%; 68% had waist circumference >80 cm; 30% >88 cm; 65% and 78% had >0.80 and ≥0.50 waist/hip index, respectively. The nurses of the night shift had the highest percentage of ABCD (87%) in comparison with nurses of the morning (80%) or evening (67%) shifts (*p* = 0.031). ABCD was associated with seniority at work (≥15 years) (*p* = 0.006), but not with the age, Table 1.

Mean weight was 68.8 ± 12.1 kg, body mass index (BMI) was 28.6 ± 4.8 kg/m^2^, waist circumference was 84.8 ± 10 cm, waist/hip index 0.54 ± 0.06, systolic blood pressure was 109.4 ± 7.7 and diastolic blood pressure was 71.4 ± 7. Only one nurse had hypertension, 25% had metabolic syndrome, 67.5% had abnormal fasting glucose. The 45% and 41% had hypercholesterolemia and hypertriglyceridemia, respectively. Hyperglycemia (*p* = 0.05), hypercholesterolemia (*p* = 0.026) and hypertriglyceridemia (*p* ≤ 0.001) were associated with ABCD (Table 2).

About the lifestyle factors explored, 42% practiced exercise, but only 3% followed the international recommendations: ≥2.5 h per week and ≥30 min in each session. Nurses who practiced physical activity less than three days per week, had risk of being ABCD (OR = 4.1) and less than 30 min in each session had also risk (OR = 2), Table 3.

On the other hand, about emotional status, 26% showed low self-esteem and 10% emotional distress. These conditions were not associated with ABCD, but low self-esteem (OR = 2, 95% CI 1.150–3.07, *p* = 0.023) and emotional distress (OR = 4, 95% CI 1.472–13.078, *p* = 0.012) were associated with unhealthy lifestyle (less of 3 days per week and/or less of 30 min per session of physical activity and poor dietary habits).

## 4. Discussion

These nurses had the highest frequency of ABCD (Ow/Ob) compared with other groups of nurses [15,16], and in comparison with the national prevalence in Mexico among women (73%) [17]. International studies have also reported high frequency of ABCD in nurses 65–76% [18], hypercholesterolemia in 30% [19], hyperglycemia in 58% [20], hypertriglyceridemia in 66% [21] and metabolic syndrome in 15–33% [22,23]; percentages slightly lower than our findings with the exception of hypertriglyceridemia and metabolic syndrome that are more frequent in other populations.

We found a higher frequency of Ow and Ob-ABCD- among night shift nurses, such as other authors have also reported over the last 30 years [24,25,26,27,28]. This means that there are still no effective strategies or these are not working for night shift nurses. On the other hand, it is important to identify that seniority at work and being a mother (having one or more children) were also associated to ABCD because nurses whit these conditions should receive more attention in order to prevent and treat Ow/Ob, since they are principal health careers for the people. Finally, working at home as a mother and outside as a nurse is a double mission that implicates less time for self-care; in fact, there is evidence that when mothers work, their children increase in weight [29], but mothers also increase their weight during the limited time they have to practice their self-care and a healthy lifestyle in the family [30].

Physical inactivity is the 4th risk factor [31] for all-cause mortality and our participants do not follow the international recommendations about doing exercise and the sedentary lifestyle is associated with chronic diseases [32]. We also need to consider the influence of urban zone in the sedentary lifestyle because nurses of a rural zone in the north of Mexico do exercise [15] at a higher proportion (63.3%) than our participants.

We found an association between low self-esteem and emotional distress with unhealthy lifestyle (sedentary and poor dietary habits), as other authors have reported [33] and in this case, despite the fact that ABCD was not associated with these psychological disorders, they may contribute to develop ABCD after a long time, so we suggest finding causality through a prospective study. On the other hand, in a study where 4980 nurses [34] were invited to participate to answer a survey, only 15.5% accepted, so then, our rate of participation was higher, however, in that study, 93% knew that ABCD needs treatment but 76% did not alert patients. It would be transcendent to achieve success among the nursing staff to prevent and treat ABCD for their own health status and also because they help physicians reinforce health counseling. If we could achieve success among the health staff it is possible we may see better results in the general population to decrease the prevalence of overweight/obesity (ABCD).

## 5. Conclusions

Nurses have a high cardiovascular risk due to the alterations found, so they must be attended immediately. Strategies to prevent ABCD should be directed at all health workers, but specially to nurses with children, seniority, working night shifts and with psychological disorders.

## Figures and Tables

**Table 1:** Demographic characteristics in ABCD vs. normal BMI.

	ABCD *n* = 211 (80)		Normal-BMI *n* = 54 (20)		
	No.	%	No.	%	*p* *
Age (years)					
23–39	76	74	27	26	0.148
40–49	102	84	19	16	
≥50	33	80	8	20	
Education level					
Technician	95	83	19	17	0.288
Specialist	17	77	5	23	
Bachelor	91	78	25	22	
Master	8	62	5	38	
Seniority					
<15 years	51	67	25	33	0.006
15–29 years	142	84	26	16	
≥30 years	18	86	3	14	
Shift					
Morning	116	80	29	20	0.031
Evening	30	67	15	33	
Night	65	87	10	13	
Service area					
Intensive care	131	78	38	22	0.526
Inpatients	41	84	8	16	
Outpatients or without patients	39	83	8	17	
Civil state					
With partner	136	82	30	18	0.526
Without partner	75	76	24	24	
Number of children					
None	46	70	20	30	0.021
1–5	165	83	24	17	

* Pearson Chi.

**Table 2:** Biochemical characteristics in ABCD vs. normal BMI.

	ABCD			Normal BMI		
	*n* = 211 (80)			*n* = 54 (20)		
	No.	%	No.	%	OR * (95% CI)	*p* ^†^
Cholesterol						
High (>200 mg/dL)	102	48	17	31	2.0 (1.1, 3.8)	0.026
Triglycerides	102	84	19	16		
High (>150 mg/dL)						
Glucose						
High (≥100 mg/dL)	99	47	10	19	3.9 (1.9, 8.1)	0.000
Index waist-hip	149	71	30	56	1.9 (1.0, 3.5)	0.056
High (cardiovascular risk)	50	76	27	50	3.2 (1.1, 1.6)	0.000

* Odds Ratio. ^†^ Pearson Chi square.

**Table 3:** Physical activity in ABCD vs. normal BMI.

Physical Activity	ABCD			Normal BMI		
	*n* = 211 (80)			*n* = 54 (20)		
	No.	%	No.	%	OR * (95% CI)	*p* ^†^
Days per week						
<3 (*n* = 257)	207	98	50	93	4.1 (1.0, 17.1)	0.095
Minutes per session						
<30 (*n* = 154)	130	62	24	44	2.0 (1.1, 3.7)	0.033

* Odds Ratio. ^†^ Pearson Chi square.
